# Employing Indirect Adenosine 2_A_ Receptors (A_2A_R) to Enhance Osseointegration of Titanium Devices: A Pre-Clinical Study

**DOI:** 10.3390/jfb14060308

**Published:** 2023-06-01

**Authors:** Maria Jesus Pacheco-Vergara, Ernesto Byron Benalcázar-Jalkh, Vasudev V. Nayak, Edmara T. P. Bergamo, Bruce Cronstein, André Luis Zétola, Fernando Pessoa Weiss, João Ricardo Almeida Grossi, Tatiana Miranda Deliberador, Paulo G. Coelho, Lukasz Witek

**Affiliations:** 1 Center for Craniofacial Molecular Biology, Herman Ostrow School of Dentistry, University of Southern California, Los Angeles, CA 90089, USA; 2 Department of Prosthodontic and Periodontology, Bauru School of Dentistry, University of Sao Paulo, Bauru 17012-901, SP, Brazil; 3 Department of Biochemistry and Molecular Biology, University of Miami Miller School of Medicine, Miami, FL 33136, USA; 4 Division of Biomaterials, New York University College of Dentistry, 345 E 24th St., Room 902D, New York, NY 10010, USA; 5 Department of Medicine, New York University Langone Medical Center, New York, NY 10016, USA; 6 Oral and Maxillofacial Surgeon, Chairman of Implantology, SOEPAR, Curitiba 80730-000, PR, Brazil; 7 Faculdade Evangélica do Paraná (FEPAR), Curitiba 80730-000, PR, Brazil; 8 School of Health Science, Universidade Positivo, Curitiba 80740-050, PR, Brazil; 9 Latin American Institute of Dental Research and Education—ILAPEO, Curitiba 80710-150, PR, Brazil; 10 Division of Plastic Surgery, Department of Surgery, University of Miami Miller School of Medicine, Miami, FL 33136, USA; 11 Department of Biomedical Engineering, New York University Tandon School of Engineering, Brooklyn, NY 11201, USA

**Keywords:** osseointegration, dipyridamole, titanium implants, low-density bone

## Abstract

The present study aimed to evaluate the effect of dipyridamole, an indirect adenosine 2A receptors (A_2A_R), on the osseointegration of titanium implants in a large, translational pre-clinical model. Sixty tapered, acid-etched titanium implants, treated with four different coatings ((i) Type I Bovine Collagen (control), (ii) 10 μM dipyridamole (DIPY), (iii) 100 μM DIPY, and (iv) 1000 μM DIPY), were inserted in the vertebral bodies of 15 female sheep (weight ~65 kg). Qualitative and quantitative analysis were performed after 3, 6, and 12 weeks in vivo to assess histological features, and percentages of bone-to-implant contact (%BIC) and bone area fraction occupancy (%BAFO). Data was analyzed using a general linear mixed model analysis with time in vivo and coating as fixed factors. Histomorphometric analysis after 3 weeks in vivo revealed higher BIC for DIPY coated implant groups (10 μM (30.42% ± 10.62), 100 μM (36.41% ± 10.62), and 1000 μM (32.46% ± 10.62)) in comparison to the control group (17.99% ± 5.82). Further, significantly higher BAFO was observed for implants augmented with 1000 μM of DIPY (43.84% ± 9.97) compared to the control group (31.89% ± 5.46). At 6 and 12 weeks, no significant differences were observed among groups. Histological analysis evidenced similar osseointegration features and an intramembranous-type healing pattern for all groups. Qualitative observation corroborated the increased presence of woven bone formation in intimate contact with the surface of the implant and within the threads at 3 weeks with increased concentrations of DIPY. Coating the implant surface with dipyridamole yielded a favorable effect with regard to BIC and BAFO at 3 weeks in vivo. These findings suggest a positive effect of DIPY on the early stages of osseointegration.

## 1. Introduction

Currently, endosteal implants are considered the gold standard to successfully rehabilitate partial and complete edentulous patients [1]. Although biological and mechanical complications are somewhat frequent, the long-term outcomes reported on clinical trials have demonstrated high survival rates for dental implants [1,2]. Nevertheless, approximately 84% of implant failures have been associated with inadequate early osseointegration [2]. Local (e.g., poor bone quality, compromised bone volume, immediate implantation, etc.) and systemic factors (e.g., systemic diseases, use of tobacco, etc.) may impair bone metabolism and have been often associated with increased risk for premature implant failure [3,4]. Therefore, early osseointegration is still considered a challenge in areas with more trabecular and low-density bone [5,6], where physicochemical modifications to the implant surface and modifications in implant macrogeometry seem to be crucial in decrease healing time while concurrently achieving and maintaining the quality and quantity of the newly formed bone surrounding the implanted device [7].

Osseointegration occurs when there is direct contact between the implant surface and the alveolar bone without any soft tissue intermediate [8], and it is mediated by some of the basic mechanisms of wound healing including the migration of inflammatory cells, deposition of extracellular matrix, and its posterior organization and remodeling [9]. Depending on the interplay between the implanted device and the osteotomy dimensions, osseointegration may occur through different healing modes [10]. When there is a tight fit between the implant and osteotomy walls, an interfacial osseointegration through bone reabsorption and bone apposition may take place. In contrast, implants with increased thread pitch and distinct thread diameters, in addition to sufficient surgical instrumentation, allows for the establishment of healing chambers between threads, the implant’s inner diameter, and osteotomy walls, which allows for intramembranous healing through the deposition of essential bone remodeling cells and proteins. Finally, a hybrid pathway may take place when the osteotomy and implant design allow for the intimate contact of the tip implant threads with native bone while allowing for the formation of healing chambers between the threads [10].

During the osseointegration process, a robust primary stability and the initial resistance of the implant to micro- or macro-motion have been considered essential to predictably achieve osseointegration [11]. Conventionally, primary stability has been associated with increased insertion torque values attained through undersized bone osteotomies. High insertion torque has the potential to yield microcrack formation and strain in the surrounding bone, which may lead to compression necrosis and bone remodeling increasing osseointegration times [12]. Therefore, several factors have been investigated in an effort to enhance primary stability and promote efficiently the achievement of secondary stability, including implant design [13], osteotomy size [14], surgical instrumentation [15], and modifications to the implant’s surface to hasten the biological interactions at the bone–implant interface [10,16,17,18,19].

Previous literature suggests that small design variations in macro- and micro-features may positively influence the stability of the implant and potentially the bone response in the early stages of osseointegration [20,21]. Modifications in several design features such as thread pitch and thickness have been assessed, aiming to enhance primary stability and avoid excessive strain in bone at implant placement [20,22,23]. Different surgical instrumentation techniques have been also the center of pre-clinical research aiming to achieve predictable osseointegration in low-density bone [24,25]. Among them, the use of non-subtractive densifying burs that promote the plastic deformation of the bone by rolling or sliding contact has evidenced promising results to enhance the implant’s primary stability and to shorten healing times for implants placed in low quality bone [26,27]. While parameters such as implant design, surface modifications, and surgical instrumentation have been studied separately, evidence suggests that the optimization of the osseointegration process may not be achieved by modifications in a single factor [10,19].

Furthermore, the use of local, sustained drug release at the bone-to-implant interface has been researched through different coatings containing osteogenic agents such as growth factors, hormones, and pharmacological agents [28,29,30]. Likewise, several techniques have been reported for chemical agent delivery to implant sites, coatings, injectable gels, microsphere hydrogel, and collagen sponges [31]. These approaches have been considered of interest to stimulate cellular response for reconstructive procedures avoiding the risks usually associated with systemic administration such as loss of drug bioavailability and high drug doses [32]. The stimulation of the cellular response at the interface has the potential to yield increased osseoconductibility, improved mineral deposition, and subsequently, expedited, predictable, long-lasting osseointegration [33,34,35].

Among osteogenic compounds, dipyridamole (DIPY), an indirect adenosine 2_A_ receptor (A_2A_R), has become of interest due to its osteoinductive properties and well-established history of safe use as an antithrombotic agent and vasodilator drug in both adult and pediatric patients [36,37]. As an antiplatelet drug, DIPY appears to act by synergistically modifying different pathways, including the inhibition of platelet cAMP-phosphodiesterase, and by the potentiation of adenosine inhibition of platelet function by blocking reuptake by vascular and blood cells. These processes have been suggested to inhibit platelet function by increasing platelet cAMP through both a reduction in enzymatic cAMP-degradation and stimulation of cAMP formation via activation of adenylcyclase by adenosine [38]. As an osteoinductive agent, DIPY increases extracellular adenosine levels by blockade of cellular purine uptake via equilibrative nucleoside transporter (ENT)-1, which stimulates osteoblast proliferation and differentiation [39,40]. Additionally, DIPY has a role in osteoclast and inflammation inhibition, which may further support the bone formation process [41]. Furthermore, DIPY has been proven to stimulate bone regeneration to levels comparable to that of growth factors such as Bone Morphogenetic Protein 2 (BMP-2), one of the most studied osteogenic agents for bone regeneration, without the associated side effects, such as ectopic bone formation, osteolysis, and craniosynostosis [36,41,42,43]. Additionally, tissue-engineering scaffolds loaded with dipyridamole have been proven to be an effective approach to enhance bone augmentation while preserving cranial suture patency for pediatric cranial reconstructions [44]. Thus, DIPY has become a material of interest to stimulate bone formation in different bone augmentation biomedical applications [43,45].

A systematic review of the literature evaluating local and sustained drug release at the bone–implant interface in different animal models revealed a positive influence of locally delivered chemical compounds during the osseointegration process [32]. While several chemical substances have been reported in previous pre-clinical trials aiming to hasten the osseointegration process in low density/quality bone (calcium phosphate, bisphosphonates, growth factors such as BMPs and hormones such as growth hormone and parathyroid hormone) [32,46,47], the ideal adjunctive osteogenic therapy to accelerate bone formation around titanium implants in challenging scenarios remains unclear. To the best of the authors’ knowledge, this is the first study to report the association of the osteoinductive properties of DIPY with endosteal titanium implants in an effort to facilitate the osseointegration process in inferior, low-density bone. Therefore, this study aimed to evaluate the influence of different DIPY coating concentrations (10, 100, and 1000 μM) on the osseointegration of titanium dental implants at 3, 6, and 12 weeks in a low-density bone translational pre-clinical model. The postulated null hypothesis was that there would be no changes with respect to the osseointegration in the DIPY groups in comparison with the control group, independent of DIPY concentration and time in vivo.

## 2. Materials and Methods

### 2.1. Surgical Model and Procedure

Prior to any surgical intervention, the team submitted the protocol for approval from the Research Ethics Committee on Animal Use (CEUA) at the Positivo University (Protocol 274/2015) in accordance with the provisions of the Arouca Law (11794/2008) and designed according to ARRIVE guidelines. After receiving approval from the committee, a total of 15 female sheep (*sp. Dorset Cruz*) ~2 years old weighing ~65 kg were acquired and allowed to acclimate for 7 days at the facility. The cervical spine of sheep was selected due to its low density and size large enough to allow the placement of all experimental groups in each subject. After an acclimatation period of one week, the surgical procedure was performed under general anesthesia. Anesthesia was induced with sodium pentothal (15–20 mg/kg) in a Normasol solution injected into the jugular vein and maintained with isofluorane (1.5–3%) in O_2_/N_2_O (50/50). Animals were monitored with ECG, SpO_2_, end tidal CO_2_, and body temperature, which was regulated by a circulating hot water blanket. The surgical area was shaved and prepared for surgery with iodine solution.

**Figure 1:**
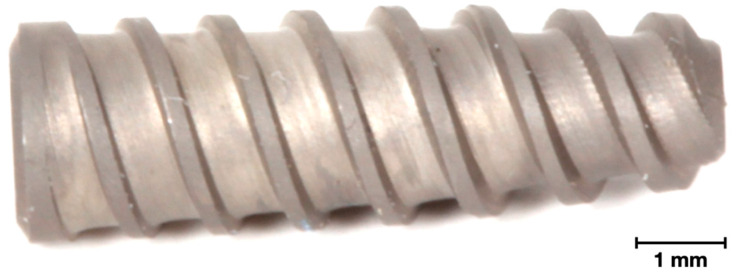
Figure depicting the design of the conical screw-type acid-etched type V Titanium alloy implant used in the current study.

**Figure 2:**
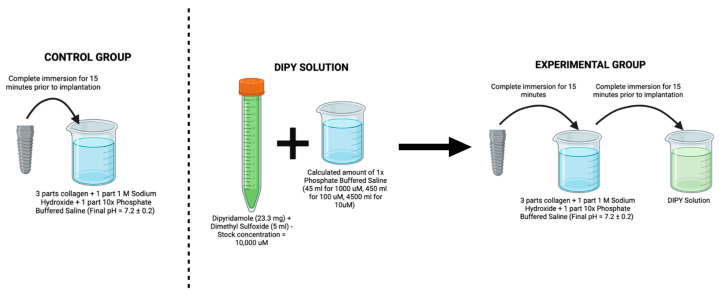
Figure depicting the coating process of the implant used in the current study (Image generated on Biorender.com (accessed on 6 April 2023).

A ~15 cm incision starting ~5 cm below the cricoid cartilage along the midline was performed for anterior access, followed by blunt dissection to access the anterior flange of the vertebrae. A conventional surgical drilling protocol for implant placement was used in a 3-step series of 2.0 mm pilot, 3.2 mm, and 3.8 mm twist drills (Emfils Colosso Drills, Itu, Brazil) under constant irrigation. Each sheep received four conical screw-type acid-etched type V Titanium alloy implants (Novo Colosso, (Diameter: 4 mm × Length: 10 mm) Emfils, Itu, Brazil) (Figure 1), which were inserted in an interpolated fashion in C3, C4, or C5 vertebral bodies, with randomized vertebrae and implant position within the vertebral body with a minimum distance of 6 mm between implants. Implants were placed bilaterally and divided as follows: one positive control group, (i) COLL, where implants were coated with bovine collagen (Collagen Type I Corning Inc., Corning, NY, USA), and three experimental groups, that in addition to the collagen coating received increasing DIPY concentrations as follows: (ii) 10 μM, (iii) 100 μM, and (iv) 1000 μM (Figure 2).

After implant placement, wound closure was achieved through simple suture with 2-0 polyglactin absorbable suture (Vicryl Ethicon, São Paulo, SP, Brazil) on the muscle’s fascia, followed by continuous skin suture with 2-0 nylon thread (Shalon Surgical Threads Ltd.a, São Luiz de Montes Belos, GO, Brazil). Ketoprofen 10% (3 mg/kg, 10% Biofen, Biofarm Química e Farmacêutica LTDA, Jaboticabal, SP, Brazil) and enrofloxacin 10% (2.5 mg/kg, injectable Chemitril 10%, Chemitec Agro Veterinária LTDA, São Paulo, SP, Brazil) were administered intramuscularly after surgery once a day for 3 days and 5 days, respectively. After surgery, food and water were given ad libitum to the animals. Five sheep were euthanized by randomization at each evaluation time point (3, 6, and 12 weeks) after surgery through a rapid intravenous injection of sodium thiopental (8 mg/mg, Thiopentax, Cristália, São Paulo, SP, Brazil) and posterior euthanization by anesthetic overdose.

### 2.2. Sample Preparation and Histomorphometric Analysis

Each implant and the surrounding bone were removed en bloc for histological processing. The vertebral blocks were dehydrated gradually in EtOH solutions ranging from 70 to 100% and embedded in MMA polymeric resin. The embedded samples were cut into ~300 μm thick sections using a slow speed diamond blade (Isomet 2000, Buehler Ltd., Lake Bluff, IL, USA) aiming at the implant’s longitudinal axis. Sections were then glued into individual acrylic slides and ground under continuous water irrigation in a grinding machine (Metaserv 3000, Buehler, Lake Bluff, IL, USA) with a series of SiC abrasive papers (400, 600, 800, and 1200) until the slides were ~100 μm thick.

Stevenel’s Blue and Van Gieson’s picro-fuchsin were used to stain the bone and soft tissue. The sections were scanned with an automated microscope and specialized computer software (Aperio Technologies, Vista, CA, USA). The digital micrographs were analyzed qualitatively and quantitatively through specific image analysis software (Image J, NIH, Bethesda, MD, USA). Percentages of bone-to-implant contact (BIC), along the total implant perimeter, and for bone area fraction occupancy (BAFO), between the implant threads, were calculated by a calibrated, single, blinded evaluator after a good intraclass correlation coefficient (between 0.9 and 1) was obtained in the inter reliability measurements.

### 2.3. Statistical Analysis

The statistical analysis was performed with IBM SPSS (v23, IBM Corp., Armonk, NY, USA), with histomorphometric data presented as mean values with 95% confidence interval values (mean ± 95% CI). %BIC and %BAFO values were analyzed with a linear mixed model with time in vivo (3, 6, and 12 weeks) and coating (COLL, 10 μM DIPY, 100 μM DIPY, and 1000 μM DIPY) as fixed factors. All values were previously assessed for normality with the Shapiro–Wilk test (*p* > 0.05).

## 3. Results

All animals recovered well from the surgery and showed no signs of complication, disease, or infection.

### 3.1. Histomorphometric Analysis

**Figure 3:**
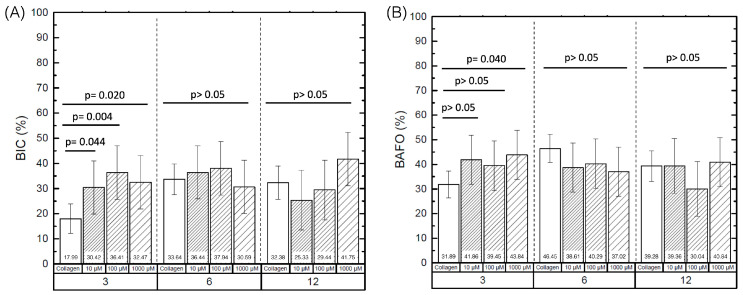
Implants coated in (i) Collagen, (ii) 10 μM DIPY, (iii) 100 μM DIPY, and (iv) 1000 μM DIPY at 3, 6, and 12 weeks, respectively. (**A**) Bone to Implant Contact (%), (**B**) Bone area fraction occupancy (%). Error bars represent 95% confidence interval (CI).

The quantitative histomorphometric analysis of BIC and BAFO between the implant groups as a function of coating and time in vivo is summarized in Figure 1. Evaluation of BIC detected statistically significant differences between control (17.99% ± 5.82) and DIPY coated groups (10 μM (30.42% ± 10.62), 100 μM (36.41% ± 10.62), and 1000 μM (32.46% ± 10.62)) at 3 weeks (*p* < 0.04) (Figure 3A). No significant differences were observed for BIC among experimental groups at six or twelve wks (*p* > 0.05).

Statistical evaluation of BAFO revealed significantly higher values at 3 weeks for implants coated with 1000 μM of DIPY (43.84% ± 9.97) compared to the control group (31.89% ± 5.46) (*p* = 0.04), with no significant differences regarding 100 (39.45% ± 9.97) and 10 μM (41.846% ± 9.97) groups (*p* > 0.05) (Figure 3B). Additionally, no significant differences were detected for BAFO between the control, 10, and 100 μM groups at 3 weeks (*p* > 0.05). Likewise, no significant differences were observed for BAFO between groups at 6 and 12 wks, independent of the DIPY coating concentration (*p* > 0.05).

### 3.2. Histological Analysis

**Figure 4:**
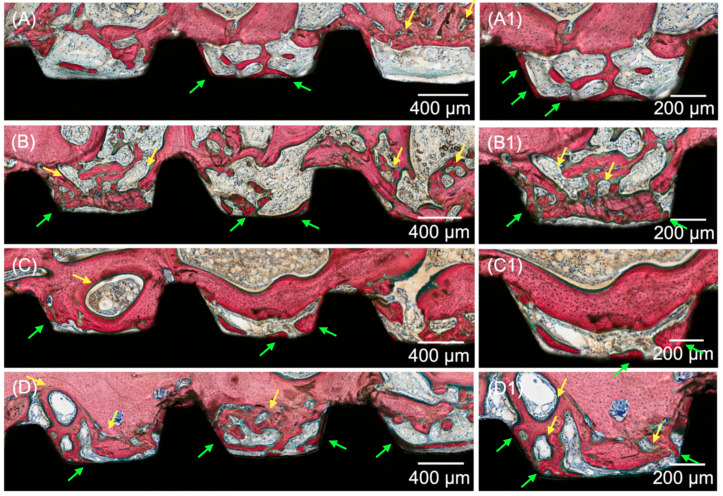
Representative optical micrographs of implant groups at 3 wks: (**A**) Collagen-coated implant, (**B**) 10 μM DIPY-coated implant, (**C**) 100 μM DIPY-coated implant, and (**D**) 1000 μM DIPY-coated implant, (**A1**–**D1**) Healing chamber and bone interface at increased magnification. Bone formation is observed to occur within the healing chambers from the surgically prepared native bone, from the implant surface (green arrows), and from the central region of the chambers, where bone remodeling sites were observed (yellow arrows).

**Figure 5:**
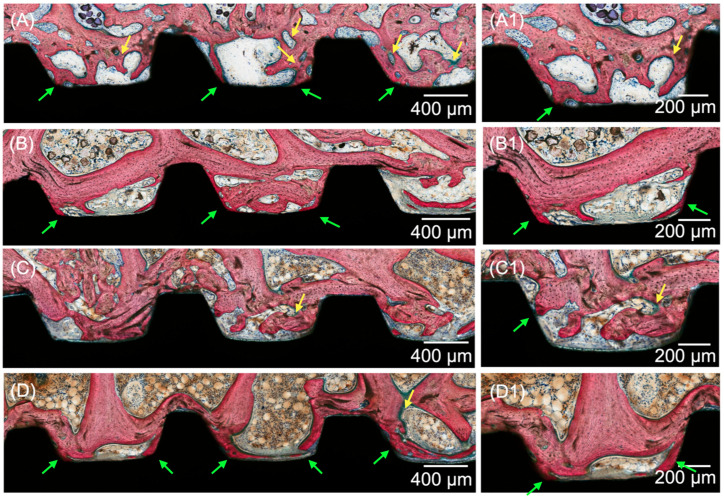
Representative optical micrographs of implant groups at 6 wks: (**A**) Collagen-coated implant, (**B**) 10 μM DIPY-coated implant, (**C**) 100 μM DIPY-coated implant, and (**D**) 1000 μM DIPY-coated implant, (**A1**–**D1**) Healing chamber and bone interface at higher magnification. Healing chamber and bone interface at higher magnification. Bone formation is observed to occur within the healing chambers from the surgically prepared native bone, from the implant surface (green arrows), and from the central region of the chambers, where bone remodeling sites can be observed (yellow arrows).

**Figure 6:**
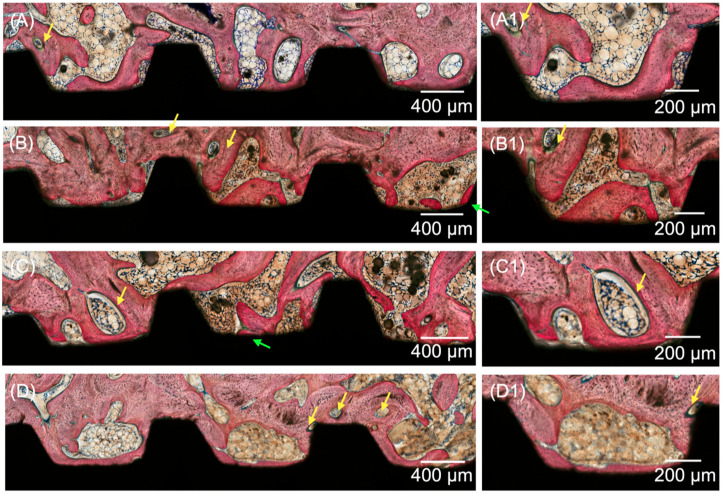
Representative optical micrographs of implant groups at 12 wks: (**A**) Collagen-coated implant, (**B**) 10 μM DIPY-coated implant, (**C**) 100 μM DIPY-coated implant, and (**D**) 1000 μM DIPY-coated implant, (**A1**–**D1**) Healing chamber and bone interface at higher magnification. Bone formation is observed to occur within the healing chambers from the surgically prepared native bone, from the implant surface (green arrows), and from the central region of the chambers, where bone remodeling sites can be observed (yellow arrows).

Histological evaluation of the micrographs at different magnifications supported the results obtained for the histomorphometric analyses. All groups yielded analogous osseointegration attributes in trabecular bone, where an intramembranous-type healing pattern was discerned at the implant healing chambers. Higher degrees of woven bone formation in intimate contact with the implant and its respective threads were observed at 3 weeks for all DIPY-coated implants when compared to the control group (Figure 4). Qualitative observation suggested the presence of similar histological features for all experimental groups (10, 100, and 1000 μM), regardless of the concentration of DIPY applied to the implant surface. At 6 and 12 weeks (wks) (Figure 5 and Figure 6, respectively), woven bone progressive substitution by lamellar bone was observed in all groups independent of the presence/concentration or absence of DIPY coating, with similar characteristics for all groups.

## 4. Discussion

The study aimed to assess the effect of varying dipyridamole coating concentrations on the osseointegration of endosteal Ti implants placed in low-density bone. The results after 3 weeks in vivo demonstrated significantly higher bone in direct contact with the implant’s surface on all experimental groups coated with DIPY compared to the control group (Type I Bovine Collagen). Significantly higher bone formation within the implant’s threads was observed for implants coated with the higher DIPY concentration (1000 μM) relative to the control group. While no significant differences were found at 6 and 12 wks in vivo between experimental and control groups, the findings at 3 wks suggest a positive effect of DIPY on the early stages of osseointegration, leading to the rejection of the null hypothesis of the present study.

The use of DIPY as an osteogenic agent has been previously explored in association with three-dimensionally printed scaffolds to promote bone regeneration in different animal models, with promising results [41,42,45]. A pre-clinical study on New Zealand White (NZW) rabbits evaluated the effect of three dimensionally printed bioceramic scaffolds loaded with different DIPY concentrations (10, 100, and 1000 μM) to treat critical-sized long bone defects, demonstrating enhanced bone formation associated with 100 and 1000 μM DIPY-loaded scaffolds relative to the lower DIPY concentration (10 μM) and control group [42]. Similar results were reported by Lopez et al. in the treatment of critical-sized mandibular defects in a rabbit model, where three-dimensionally printed bioceramic scaffolds augmented with 100 μM DIPY yielded increased bone formation across the defect relative to the group treated without DIPY [45]. Furthermore, a sheep study that evaluated bone regeneration in calvarial defects demonstrated that loading 3D-printed bioceramic scaffolds with 100 μM of DIPY significantly enhance the scaffolds’ osteogenic properties compared to non-loaded groups [41]. The increased bone formation in the experimental groups of the aforementioned studies has been attributed to the ability of DIPY to blockage Ent1 transporter, increasing extracellular adenosine levels, and promoting new bone formation through the stimulation of osteoblast differentiation and the blockage of osteoclast differentiation and function [40]. This mechanism may also explain the significant increase in osseointegration parameters at 3 weeks observed in the present study, which suggests a positive effect of DIPY to hasten initial bone healing after implant placement.

While similar concentrations have been used in the present work, the application of DIPY as a coating material of dense titanium implants presents a healing scenario that differs significantly than the one provided by the use of porous bioceramic scaffolds for bone regeneration. In the former scenario, it is likely that the dose is absorbed within the early stages, which may explain the significant effect of DIPY at 3 weeks and the absence of differences between experimental and control groups after 6 and 12 weeks. Similar results have been observed in previous studies where titanium implants were coated with different osteogenic chemical compounds (such as calcium phosphate, bisphosphonates, and bone morphogenetic proteins), with experimental groups demonstrating higher values of BIC regarding control groups [32]. For instance, a pre-clinical study in sheep that evaluated coating of dental implants with the osteogenic agent recombinant human bone morphogenetic protein 2 (rhBMP-2) demonstrated a significant increase in osseointegration parameters at 3 weeks, with no significant effect at 6 weeks in vivo [35], which is in agreement with the present study.

The osteogenic effects of DIPY and rhBMP-2 for bone regeneration have been previously compared in pre-clinical studies [43,48]. Ishack et al. demonstrated that 100 μM DIPY-coated β-Tricalcium Phosphate/Hydroxyapatite scaffolds were as effective as 200 ng/mL BMP-2-coated scaffolds in critical sized bone defects in mice [48]. Lopez et al. reported that 1000 μM and 10,000 μM DIPY-loaded 3DPBC scaffolds were as effective in regenerating vascularized bone as rhBMP-2 (0.2 g/mL)-loaded 3DPBC scaffolds on NZW rabbits with 3.5 mm × 3.5 mm alveolar resection adjacent to the growing suture [43]. These studies have demonstrated the effectiveness of DIPY as an osteogenic agent, with the advantage of averting the side effects caused by rhBMP-2 administration such as risk of osteolysis and ectopic bone formation [36,41,42]. Furthermore, recent literature has suggested that seeding β-TCP scaffolds with osteogenic agents may accelerate the degradation of the scaffolds, possibly because of increased vascularization promoting degradation via hydrolysis from tissue fluids [49]. Considering the use of DIPY for coating titanium implants, increased vascularization might be desirable to accelerate the interactions that take place in the implant-host interface, which may allow for increased cell adhesion to the implant surface in the early stages of osseointegration.

With respect to the use of titanium devices, the application of adjunctive therapies to stimulate bone formation in low-density and poor-quality bone has been suggested to promote faster and predictable osseointegration, potentially leading to reduced treatment time frames and earlier functional loading. Several substances have been used in previous literature aiming to stimulate bone formation or to produce local mineralization of bone surrounding dental implants at the moment of implantation in low-density bone scenarios. For instance, previous pre-clinical research in sheep presented the application of different doses of growth hormone and parathyroid hormone applied directly to the implant surface prior installation in low-density bone. Interestingly, both studies presented no significant differences in BIC and BAFO [46,47]. In a previous in vitro drug release profile study, it was reported the use of a thermo-sensitive hydrogel composite drug delivery system for the administration of DIPY. While merely in vitro characterizations and cell viability tests were performed, the authors concluded that the prepared drug delivery system might have great potential in promoting the regeneration of bone defects [50].

The sheep model, the animal model used in the present study, is a large preclinical model that has been previously used to assess differences on the osseointegration process of dental implants with diverse features [51,52]. Additionally, the consistent biologic outcomes reported in the literature, of comparable bone remodeling potential in comparison to humans and large anatomic size, enough to allow for the installation of standard-size dental implants, make sheep a reliable and well-documented model for evaluating osseointegration, especially in low-density bone [53,54,55]. Moreover, to the best of the authors’ knowledge this is the first study to report the application of DIPY as a coating material for titanium dental implant placement in low-density bone. While it was evident that DIPY enhanced early bone formation around the implanted devices, further investigations are warranted to determine the release profile of DIPY, the minimal effective doses required to efficiently promote bone formation in low-density/quality bone and to optimize implant treatment in challenging scenarios.

## 5. Conclusions

Coating the implant surface with dipyridamole increased BIC and BAFO at 3 weeks in vivo. These findings suggest a positive effect of DIPY on the early stages of osseointegration, although no significant effect of DIPY was observed in later evaluation time-points.

## Data Availability

The data that support the findings of this study are available on request from the corresponding author.
